# ﻿Four new terrestrial earthworm species from the northeast Thailand (Oligochaeta, Megascolecidae)

**DOI:** 10.3897/zookeys.1176.106517

**Published:** 2023-08-28

**Authors:** Ratmanee Chanabun, Anuwat Aoonkum, Teerapong Seesamut, Ueangfa Bantaowong, Somsak Panha

**Affiliations:** 1 Program in Animal Science, Faculty of Agricultural Technology, Sakon Nakhon Rajabhat University, Sakon Nakhon 47000, Thailand; 2 Biodiversity and Utilization Research Unit, Center of Excellence in Modern Agriculture, Sakon Nakhon Rajabhat University, Sakon Nakhon 47000, Thailand; 3 Faculty of Agricultural Technology, Sakon Nakhon Rajabhat University, Sakon Nakhon 47000, Thailand; 4 Department of Biology, Faculty of Science, Rangsit University, Pathum Thani, 12000, Thailand; 5 Division of Biology, Faculty of Science and Technology, Rajamangala University of Technology Thanyaburi, Pathum Thani 12110, Thailand; 6 Animal Systematics Research Unit, Department of Biology, Faculty of Science, Chulalongkorn University, Bangkok 10330, Thailand

**Keywords:** *Amynthas*, *Metaphire*, new species, taxonomy, Thailand

## Abstract

Earthworm specimens collected from Sakon Nakhon and Nakhon Phanom, northeast Thailand, were found to contain four new species in the family Megascolecidae, with one species in the genus *Metaphire* Sims & Easton, 1972, and the other three in the genus *Amynthas* Kinberg, 1867. These are herein named *Metaphiresongkhramensis* Chanabun & Panha, **sp. nov.** in the sexthecal *houlleti* species group, and *Amynthassakonnakhonensis* Chanabun & Panha, **sp. nov.**, *A.auriculus* Chanabun & Panha, **sp. nov.**, and *A.bantanensis* Chanabun & Panha, **sp. nov.** in the sexthecal *aelianus* species group. *Metaphiresongkhramensis* Chanabun & Panha, **sp. nov.** occurs in dark clay soil of the oxbow lake of the river, *Amynthassakonnakhonensis* Chanabun & Panha, **sp. nov.** occurs in wetland area, *A.auriculus* Chanabun & Panha, **sp. nov.** occurs in dark sandy loam habitats of mixed deciduous forest while the following species, *A.bantanensis* Chanabun & Panha, **sp. nov.** occurs in sandy loam habitats of paddy fields. Descriptions of the new species, including illustrations of the external and internal morphological characteristics, are provided.

## ﻿Introduction

Earthworms are common macro-soil invertebrates found in almost all parts of the world, in all types of habitats, such as terrestrial, aquatic, and semi-aquatic habitats. Earthworms play significant roles in the physical, chemical, and biological properties of soil (Edwards and Arancon 2022). As permanently soil-dwelling animals, their activities affect soil properties and create suitable habitats for other smaller soil flora and fauna. Moreover, earthworms can be used as bioindicators of the relative health of soil ecological systems (Fründ et al. 2011). There are approximately 6,000 species that have been recorded worldwide, and the estimated total global species diversity exceeds 8,000 species (Edwards and Bohlen 1996; Fragoso et al. 1999; Jeratthitikul et al. 2017).

Earthworms are known to decompose organic waste from houses and agricultural farms. They can improve the soil property in ecosystems by producing vermicompost and vermicompost tea that including micro-organisms without pathogenic bacteria (Wongsaroj et al. 2021), as shown for *Eiseniaandrei* Bouché, 1972, *Eiseniafetida* (Savigny, 1826), *Dendrobaenaveneta* (Rosa, 1886), *Perionyxexcavatus* Perrier, 1872, and *Eudriluseugeniae* (Kinberg, 1867) species. All these five species are widely cultivated due to their tolerance to a wide range of environmental conditions and have short life cycles, high reproductive rates, and good composting rates (Domínguez 2018; Heuzé et al. 2020).

In addition, humans have long used earthworms as a healthy diet or for medication and as a source of feed for other animals (Edwards and Bohlen 1996). Earthworm meal has plenty of nutrients and enzymes, which can help break down food and repair body tissue (Paoletti et al. 2003; Grdiša et al. 2009, 2013; Iannucci et al. 2009; Bamidele et al. 2016; Sun and Jiang 2017; Musyoka et al. 2018) as in several countries, including Japan, Korea, Taiwan, Myanmar, Laos, India, Singapore, South America, North America, Papua New Guinea, Australia, New Zealand and China (Price 1901; Benham 1904; Gates 1926; Carr 1951; Reynolds and Reynolds 1972; Paoletti and Dreon 2005; Grdiša et al. 2009; Cooper et al. 2012a, b; Sun and Jiang 2017).

**Figure 1.**
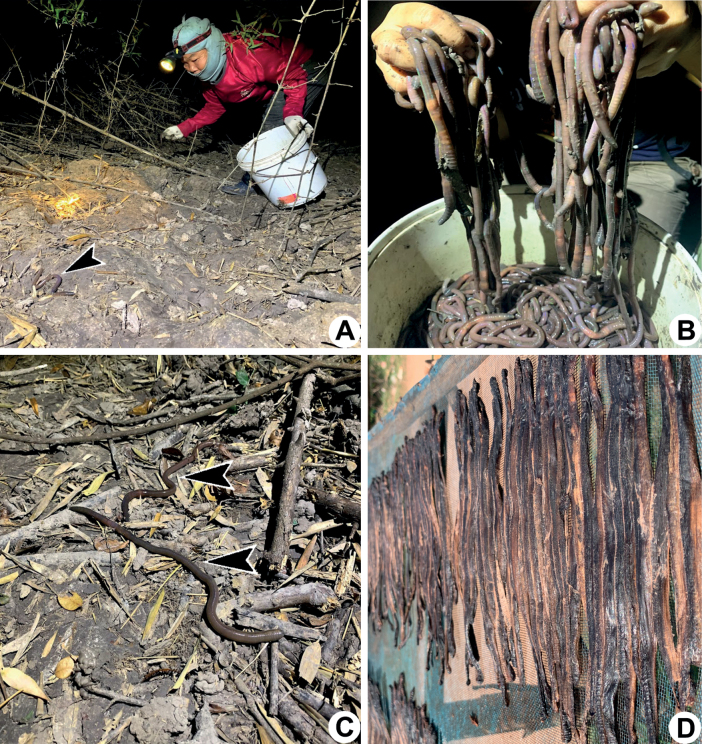
Collecting earthworms in the early morning of the winter season **A** villagers in Sakon Nakhon collect the migratory earthworms with their bare hands **B** large numbers of caught earthworms being sold to middlemen **C***Metaphiresongkhramensis* sp. nov. from Akat Amnuai, Sakon Nakhon, moving on the soil during the migration season, and **D** dried earthworms.

Economically, most of the dried earthworms from Sakon Nakhon and Nakhon Phanom, both in northeast Thailand, are exported to countries like Hong Kong, China, and Taiwan, where a large number of earthworms are consumed per year as part of traditional diet and medicine. This results in a high economic return for the villages of the mentioned provinces. At the end of the rainy season and the onset of the cold season from August to December, the villagers will go out at night and early in the morning, at approximately 2.00–5.00 a.m., to paddy fields, meadows, lowland areas, freshwater islands, or to lake shores to collect with their bare hands when fresh earthworms crawling on the soil surface. In the past, after villagers collected earthworms from the field, they would wash them in water and then rewash them in water-soaked with Burmese rosewood bark (*Pterocarpusmacrocarpus*) to discard the mucus. The earthworms were then dissected from the anterior to the posterior and completely sun-dried before selling them to the middlemen. Nowadays, the locals sell the fresh earthworms to a middleman who lives in Ban Tan, Nakhon Phanom. This province is a popular marketplace for dried earthworms. The middleman is now responsible for dissecting the earthworms with a machine and drying them in the sun. The price of dried earthworms ranges from 450–750 Thai baht/kg (14–23 USD/kg as of this writing), and that for fresh earthworms ranges from 20–45 Thai baht/kg (0.59–1.3 USD/kg as of this writing) (Fig. 1). Because of the aforementioned importance of earthworms in agriculture, food, traditional medicine, and their economic value, there is a vital need to know about earthworm species, their behaviors, habitats, and distribution ranges, in order to assure their future conservation and sustainable use. However, although Thailand sells dried earthworms, none of the species have been identified. In this paper, we report on the earthworm species that Thai people sell, and all four species described herein were from northeast Thailand and are newly discovered.

## ﻿Material and method

**Figure 2.**
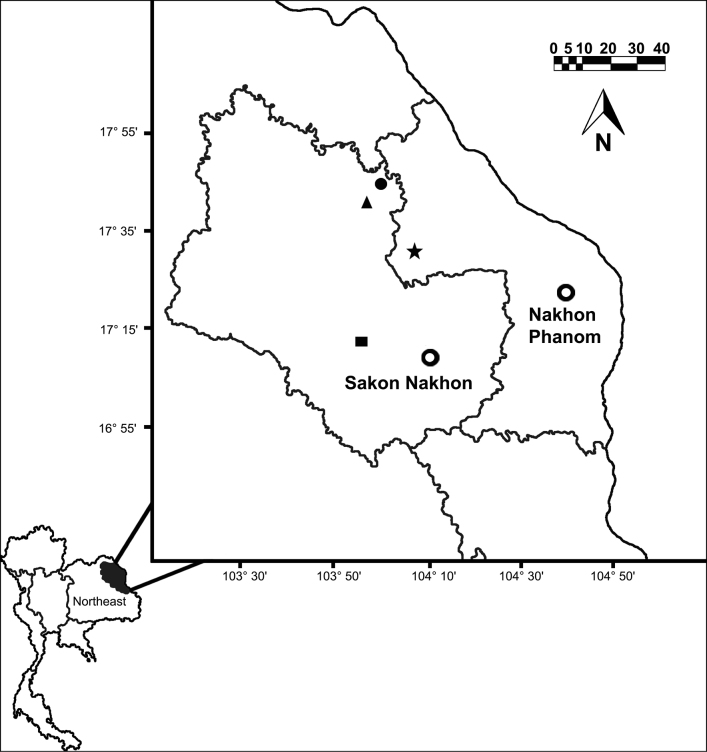
Map of the collecting localities of the new species described in this paper from northeast Thailand. Filled circle: *Metaphiresongkhramensis* sp. nov. from Kong Ngong, Akat Amnuai, Sakon Nakhon, Thailand. Filled triangular: *Amynthassakonnakhonensis* sp. nov. from Nong Tuet, Samakkee Pattana, Akat Amnuai, Sakon Nakhon, Thailand. Filled square: *Amynthasauriculus* sp. nov. from Wut Tham Kham, Phannanikom, Sakon Nakhon. Filled star: *Amynthasbantanensis* sp. nov. from Ban Tan, Nawah, Nakorn Phanom, Thailand.

Earthworm specimens from Kong Ngong from Sakon Nakhon were collected with the villagers at approximately 2.00–5.00 a.m. by hand when earthworms were crawling on the soil surface during the cold season (November). Specimens from Nong Tuet and Wut Tham Kham from Sakon Nakhon and Ban Tan from Nakhon Phanom were collected by digging and hand-sorting. All earthworm specimens are from northeastern Thailand (Fig. 2). The collected specimens were washed and killed in 30% (v/v) ethanol before being photographed and then transferred to 95% (v/v) ethanol for preservation and subsequent morphological studies. The anatomical and morphological observations were made with an ACCU-SCOPE 3075 stereo microscope. Illustrations included the body segments, distinct external characters, and internal organs. The type series are deposited in Chulalongkorn University, Museum of Zoology, Bangkok, Thailand (**CUMZ**). Additional paratypes will be deposited in the Natural History Museum (**NHMUK**), London, and at the Biozentrum Grindel und Zoologisches Museum, University of Hamburg (**ZMH**). Specimens used in this study strictly followed the protocols approved by the Institutional Animal Care and Use Committee of Khon Kaen University (IACUC-KKU) under approval number IACUC-KKU-32/65.

The following abbreviation used in the figures of the anatomy are as appeared in Bantaowong et al. (2011a, b): **sp**, spermathecal pores; **fp**, female pore; **gm**, genital markings; **mp**, male pores; **sc**, spermathecae; **sv**, seminal vesicles; **pg**, prostate gland; **ic**, intestinal caeca.

## ﻿Taxonomy

### ﻿Family Megascolecidae Rosa, 1891

**Genus *Metaphire* Sims & Easton, 1972**

#### ﻿ Metaphire songkhramensis

Taxon classificationAnimaliaCrassiclitellataMegascolecidae

Chanabun & Panha sp. nov.

64AC7215-4428-5B71-980C-69F761E41954

https://zoobank.org/5E9E4FCF-FF48-4C38-9ED6-3A89627DC625

**Figure 3.**
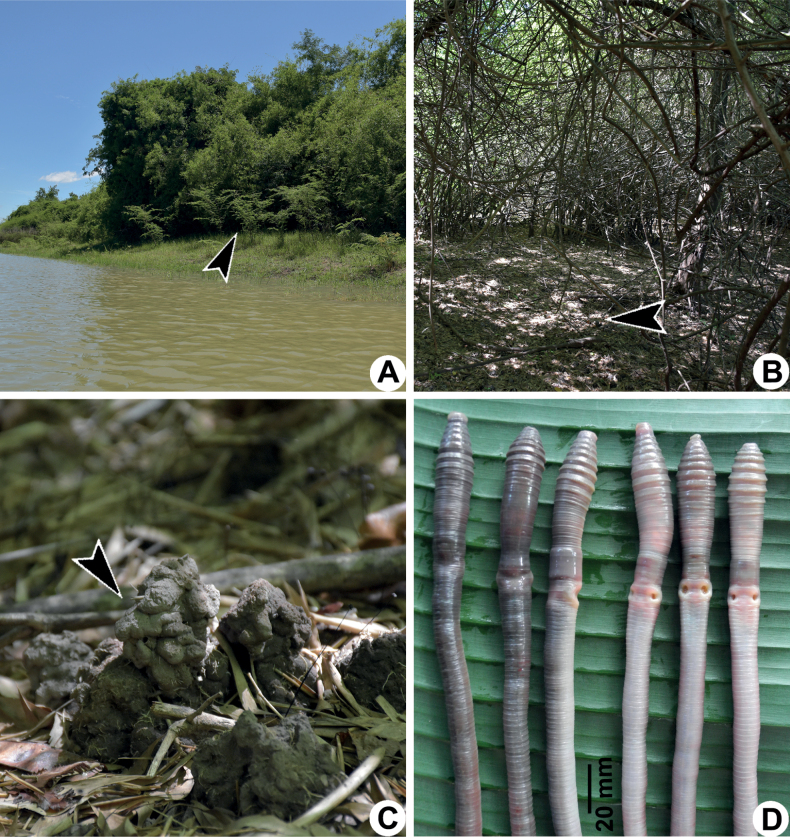
Photographs showing the **A** type locality of *Metaphiresongkhramensis* sp. nov. from Kong Ngong, Akat Amnuai, Sakon Nakhon, Thailand **B** habitat of the new species covered with small bamboo and small shrubs **C** casts of the new species, and **D** coloration of newly collected paratype (CUMZ 3822) after the first preservation step in 30% (v/v) ethanol.

**Figure 4.**
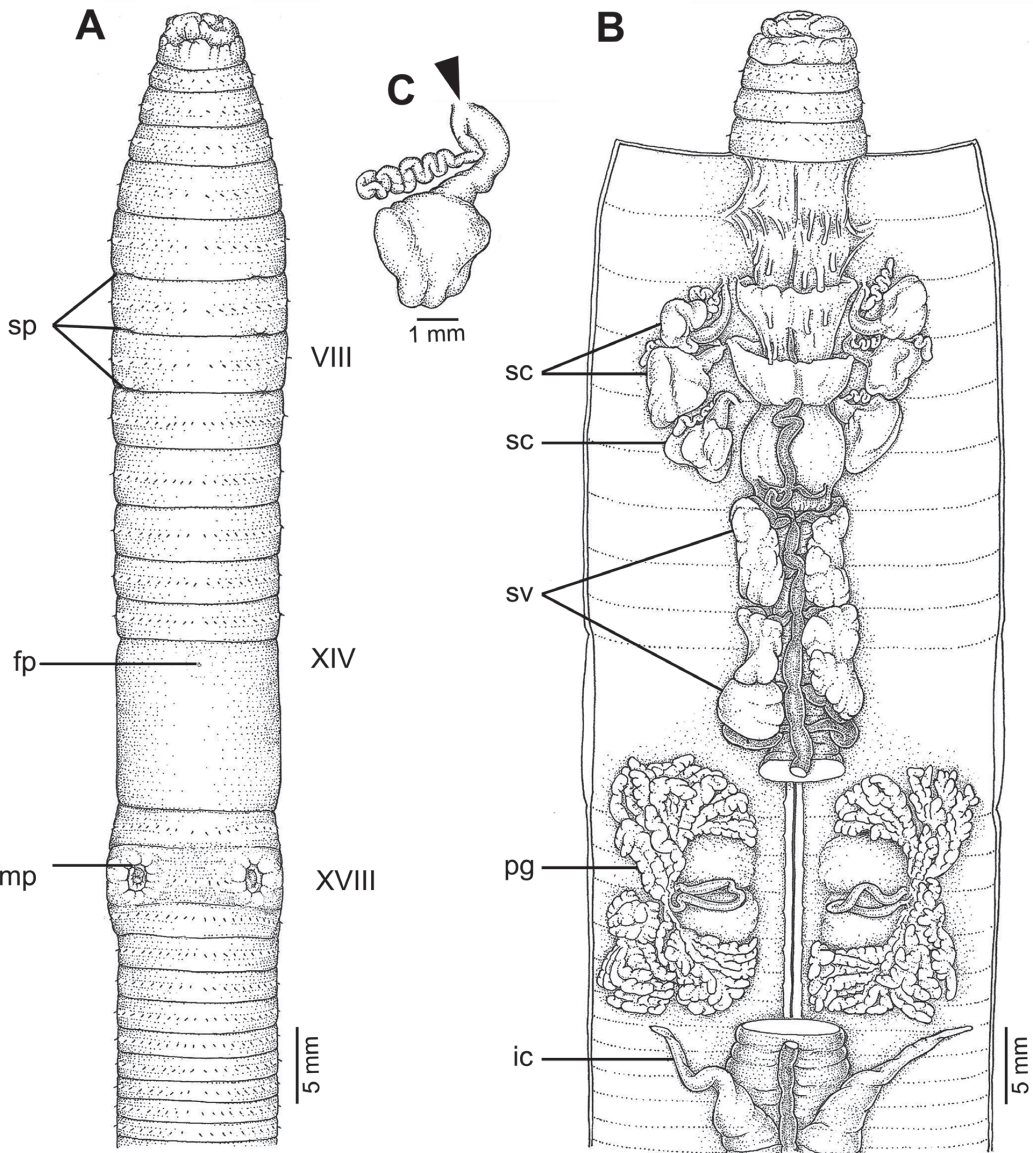
External and internal morphology of *Metaphiresongkhramensis* sp. nov., holotype (CUMZ 3821) **A** external ventral view **B** internal dorsal view, and **C** spermatheca with location of spermathecal pore arrowed.

Figs 1C , 3D , 4 , Table 1

##### Type material.

***Holotype***: Adult specimen (CUMZ 3821), Kong Ngong, Akat Amnuai, Sakon Nakhon, northeast of Thailand, 17°45'19.4"N, 104°01'18.8"E, 148 m a.m.s.l., 8 November 2022, coll. R. Chanabun, A. Aoonkum. ***Paratypes***: 45 adults (CUMZ 3822), 2 adults (NHMUK), 2 adults (ZMH), same collection data as for holotype.

##### Diagnosis.

Large sized; length 229–427 mm, diameter 10–14 mm, 129–240 segments. Male pores paired in segment XVIII, each represented by a large invaginated area, conspicuous, deep holes that resemble eyes, genital markings absent. Spermathecal pores paired in intersegment 6/7–8/9. Spermathecae with large, round, and flat ampulla, with a long and thick duct, diverticulum thin, long, and zigzagged towards the distal end. No nephridia on the spermathecal duct. Holandric. Intestinal caeca simple. First dorsal pore in 12/13. Prostate gland large in XV–XXII, long, slender with U-shape duct, large paired of glandular masses on the copulatory sac, no genital marking glands.

##### Description of holotype.

Length 333 mm, diameter 12 mm, cylindrical body with 167 segments. Preserved specimens are dark brownish on the dorsal part and pale gray on the ventral part. Setae regularly distributed around segmental equators, numbering 65 at segment VII, 85 at segment XX, and nine between male pores at segment XVIII. Setal formula AA:AB:ZZ:ZY=1:1:2:1 at segment XIII. Single female pore on the ventral side at segment XIV. Prostomium epilobic. First dorsal pore in 12/13. Clitellum annular in XIV–XVI with no dorsal pores or setae.

Male pores in copulatory pouches that have wrinkled and convex margins on ventrolateral sides of segment XVIII; pouches 5 mm apart, which is 0.13× the body circumference. These pores are large, conspicuous, deep holes that resemble eyes. There is no skin folding, and genital markings absent. Large spermathecal pores arranged in three pairs in ventral region 6/7–8/9, distance between each pair is ~ 0.26× the body circumference ventrally apart. There are no genital markings in this area.

The septa at 5/6–7/8 thick, absent in 8/9–9/10, slightly thick in 10/11–11/12, thin in 12/13–14/15, and very thin behind 15/16. Intestine begins at segment XV. Gizzard large within IX–X. Long and simple intestinal caeca in XXVII–XIX. Esophageal hearts four pairs in X–XIII. Holandric; testes and funnels in segments X and XI. Seminal vesicles are paired, one at XI–XII, the other at XIII–XIV; the hindmost pair is larger. Prostate glands large, located in segments XV–XXII, and divided into several lobules. Prostate duct long, slender with U-shape, and with large paired glandular masses on the copulatory sac at segments XVII–XVIII and XIX–XX.

The ovaries are located in segment XIII. Three pairs of spermathecae present on VII–IX. Ampulla large, round, and flat-shaped, with long and thick duct that can be clearly seen from the ampulla. Diverticulum is thin, long, and zigzagged towards the distal end.

##### Variation.

Forty-nine paratypes ranged in size from 229–427 mm (318.75 ± 40.32 mm) body length with 129–240 (159.29 ± 19.77) segments.

##### Etymology.

This new species was named after its type locality, Songkhram River.

##### Distribution.

This species is known only from the type locality.

##### Remarks.

*Metaphiresongkhramensis* is a new species of sexthecal earthworm without postclitellar genital markings. It has three pairs of spermathecal pores in intersegmental furrows 6/7–8/9. This species keys to the *houlleti* species group, which has more than 40 species (Sims and Easton 1972). Below, comparison of the new species with other species found in different regions in the *houlleti* species group: *M.umbraticola* (Gates, 1932) and *M.quadrigemina* (Gates, 1932) from Myanmar, *M.amplectens* (Michaelsen, 1934), *M.dawydovi* (Michaelsen, 1934), *M.acampanulata* Nguyen, 2022 from Vietnam (Nguyen et al. 2022), *M.bindjeyensis* (Michaelsen, 1899) from Sumatra, and *M.hijauensis* Ng & Panha, 2018 from Malysia (Ng et al. 2018) are stated.

*Metaphiresongkhramensis* sp. nov. differs from *M.umbraticola* and *M.quadrigemina* from Myanmar by having smaller size than the new species. *Metaphireumbraticola* which has a body size that ranges from 115–122 mm, a diameter of 6–7 mm, with 125–135 segments, *M.quadrigemina* which has a size that ranges from 64–72 mm by 3–4 mm, with 115, while the new species ranges from 229–427 mm, a diameter of 10–14 mm, with 129–240 segments.

*Metaphiresongkhramensis* sp. nov. differs from *M.amplectens*, *M.dawydovi*, and *M.acampanulata* from Vietnam through the body size and spermathecae. *Metaphiresongkhramensis* sp. nov. differs from *M.amplectens* by *M.amplectens* has a smaller body size than the new species (body length 44–52 mm, diameter 2½–3½ mm with 90–112 segments). *Metaphiresongkhramensis* sp. nov. differs from *M.dawydovi* in that *M.dawydovi* has a smaller size (body length 275 mm, diameter 7 mm, with 160 segments), and flask-shaped ampullae with small diverticulum, whereas the new species has a larger body size with large, round, and flat-shaped ampullae with thin, long, and zigzag diverticulum. *Metaphiresongkhramensis* sp. nov. differs from *M.acampanulata* from Vietnam in body size and spermathecae; the latter has a smaller size than the new species (body length 77–198 mm, diameter 4.03–6.91 mm with 56–144 segments), and ampulla mushroom-shaped with grooves on the surface while the new species has a larger size and has large, round, and flat-shaped of ampulla.

*Metaphiresongkhramensis* sp. nov. similar to *M.bindjeyensis* from Sumatra in body size length but *M.bindjeyensis* obclavate ampullae with zigzag diverticulum, while the new species has a large, round, and flat-shaped ampulla with a thin, long, and zigzagged diverticulum. *Metaphiresongkhramensis* sp. nov. differs from *M.hijauensis* from Malysia by the latter species has a smaller size than the new species (body size range 66–87 mm, diameter of 2.8–3.1 mm, with 101–120 segments), and the first dorsal pores in 10/11 while the new species has first dorsal pores in 12/13.

In Thailand, five species have been reported, *M.houlleti* (Perrier, 1872), *M.virgo* (Beddard, 1900), *M.perichaeta* (Beddard, 1900), *M.khaochamao* Bantaowong & Panha, 2016, and *M.khaoluangensis* Bantaowong & Panha, 2016. *Metaphiresongkhramensis* sp. nov. can be distinguished from this group of five as follows: *M.houlleti* and *M.virgo* have first dorsal pores in 11/12 while the new species has first dorsal pores in 12/13, and a large, round, and flat-shaped ampulla with a thin, long, and zigzagged diverticulum (spherical and small sac in *M.houlleti* and *M.virgo*, respectively). The new species does not have the contorted diverticulum stalk enveloped in connective tissue as found in *M.houlleti*, and also lacks the typhlosole present in *M.houlleti*. *Metaphirevirgo* has a spermathecal diverticulum stalk with multiple folds, while the new species is absent. *Metaphirehoulleti* and *M.virgo* have genital markings bearing stalked glands in association with spermathecae and copulatory sacs, while the new species lacks them. This new species differs from *M.perichaeta* in that *M.perichaeta* has a smaller size, inverted pear-shaped spermathecae with coiled diverticulum, and the last hearts in XII (Beddard 1900; Stephenson 1932). *Metaphiresongkhramensis* sp. nov. differs from *M.khaochamao* and *M.khaoluangensis*, through the male opening, body size, and spermathecae. *Metaphirekhaochamao* has smaller body size (body size range 100–148 mm, with 110–120 segments), the male field including the lateral slits associated with the male pores, the absence of setae between male pores, and an elliptic ampulla with a short duct. *Metaphirekhaoluangensis* has smaller body size (body size range 130–265 mm, with 113–131 segments), a secondary male opening with puckered margin, an elongate ampulla, and a sac-like duct, whereas the new species has a larger size with setae between male pore, a large, round, and flat-shaped ampulla, and a thin, long, and zigzag diverticulum. A comparison of characters between *M.songkhramensis* sp. nov. and other related species is presented in Table 1.

**Table 1.** Morphological characteristics of the *Metaphirehoulleti* species group in Thailand. The comma is used to separate body length and width. Data for *M.khaoluangensis* Bantaowong & Panha, 2016, *M.khaochamao* Bantaowong & Panha, 2016, and *M.perichaeta* (Beddard, 1900) are from Bantaowong et al. (2016), *M.houlleti* (Perrier, 1872) are from Gates (1972), and *M.virgo* (Beddard, 1900) are from Stephenson (1932).

Characters	*M.songkhramensis* sp. nov.	*M.khaoluangensis*	*M.khaochamao*	*M.perichaeta*	*M.virgo*	*M.houlleti*
Body length, width (mm)	229–427, 10–14	220, 10	166, 6	160, 5	152–157, 5	92–200, 4–7
Segment number	129–240	119	118	118	129	92–140
First dorsal pore	12/13	12/13	12/13	12/13	11/12	11/12
Spermathecae	large, round, flattened	elongate	elliptic	pear shape	small	large sac
Diverticulum	thin, long	slender	slender	zigzag	tubular	looped
Prostate gland	XV–XXII	XVI–XIX	XVII–XXIII	XVII–XIX	XVII–XVIII	XVI–XXI
Genital marking gland	absent	absent	absent	absent	absent	stalk
Copulatory sac	present	present	absent	present	present	present
Intestinal caeca	XXVII–XIX	XXVII–XXI	XXVII–XXIII	XXVII–XXIV	XXVII–XXV	XXVII–XXII
Type locality	Thailand	Thailand	Thailand	Thailand	Thailand	India

### ﻿Genus *Amynthas* Kinberg, 1867

#### ﻿ Amynthas sakonnakhonensis

Taxon classificationAnimaliaCrassiclitellataMegascolecidae

Chanabun & Panha, sp. nov.

8E9D8C56-CD97-54D8-A295-7C1E7893D56E

https://zoobank.org/361CB72B-D59A-47E3-90BC-B7945E33F3BD

**Figure 5.**
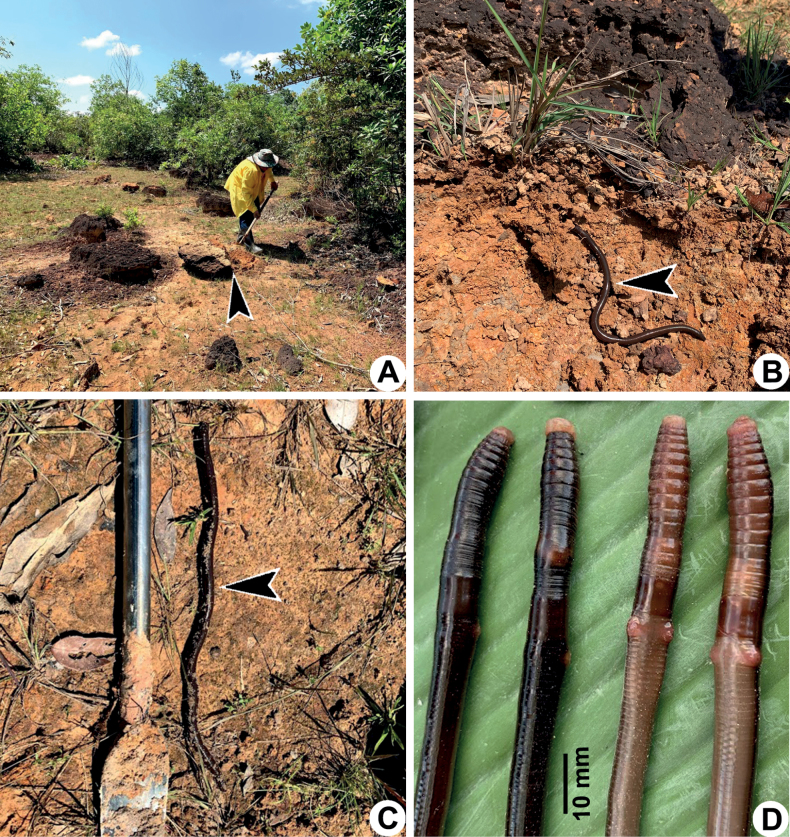
Photographs showing the **A** type locality of *Amynthassakonnakhonensis* sp. nov. from Nong Tuet, Samakkee Pattana, Akat Amnuai, Sakon Nakhon, Thailand **B** living specimen under the stone near casts **C** coloration of a living paratype, and **D** coloration of a newly collected paratype (CUMZ 3824) after the first preservation step in 30% (v/v) ethanol.

**Figure 6.**
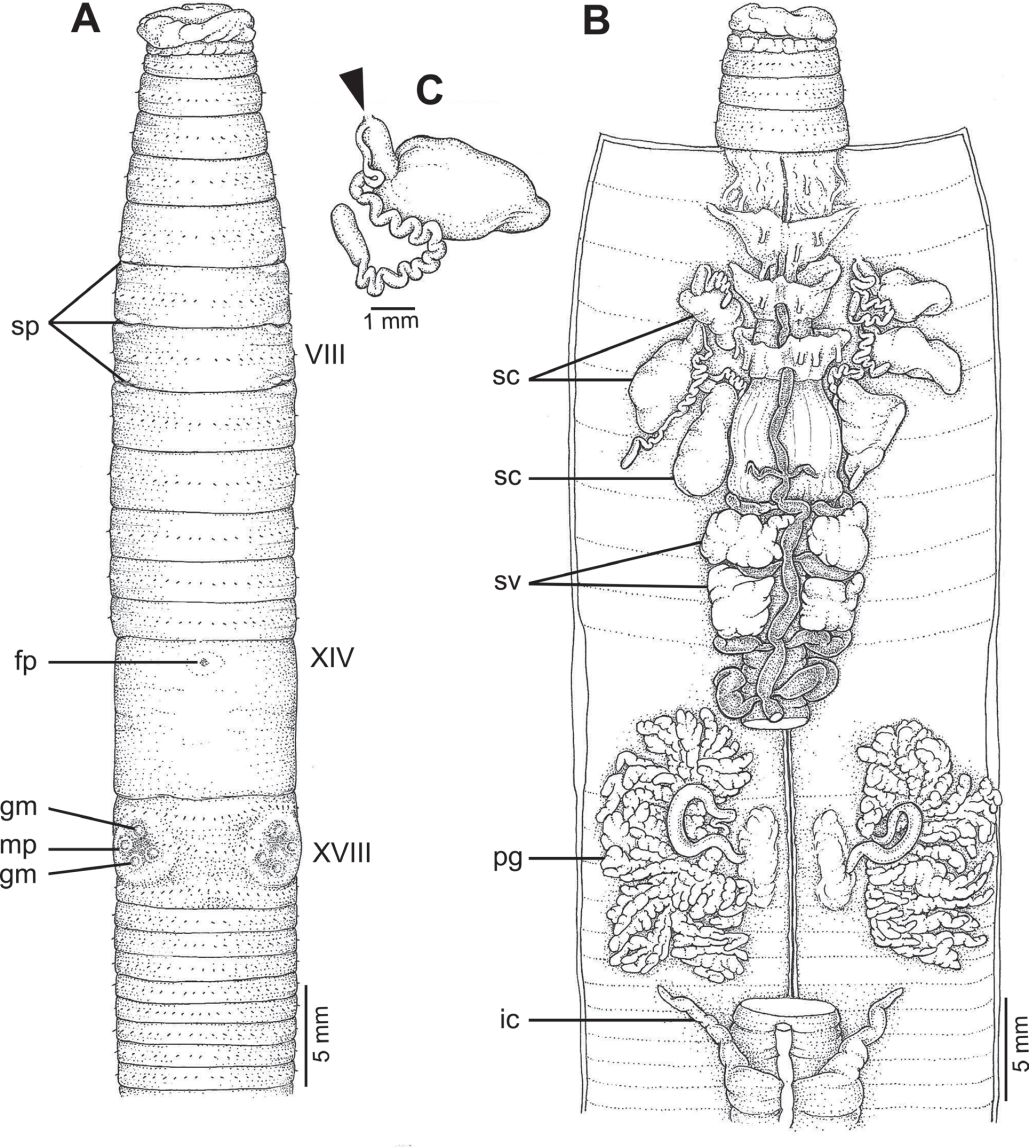
External and internal morphology of *Amynthassakonnakhonensis* sp. nov., holotype (CUMZ 3823) **A** external ventral view **B** internal dorsal view, and **C** spermatheca with location of spermathecal pore arrowed.

Figs 5 , 6 , Tables 2 , 5

##### Type material.

***Holotype***: Adult specimen (CUMZ 3823), Nong Tuet, Samakkee Pattana, Akat Amnuai, Sakon Nakhon, northeast of Thailand, 17°40'54.1"N, 103°59'50.6"E, 149 m a.m.s.l., 6 October 2019, coll. R. Chanabun, A. Aoonkum. ***Paratypes***: 25 adults (CUMZ 3824), 2 adults (NHMUK), 2 adults (ZMH), same collection data as for holotype.

##### Diagnosis.

Medium-large size, length 134–238 mm, diameter 6–9 mm, 85–162 segments. Male pores paired in segment XVIII, each surrounded by four genital markings. Paired spermathecal pores in intersegments 6/7–8/9. Spermathecae large oval sacs of the ampulla, with stout and short duct, it is less than ampullar in length. Diverticulum long and zigzagged at the beginning and dilated towards the distal end, a baton-like chamber. Intestinal origin at XV. Intestinal caeca simple. Holandric. First dorsal pore in 12/13. Prostate glands large in segments XV–XXII, its ducts long and slender, surrounded by a large, long sessile glandular mass on the body wall.

##### Description of holotype.

Length 217 mm, diameter 9 mm, body cylindrical with 156 segments. Preserved specimens are dark brownish on the dorsal part and pale gray on the ventral part. Setae regularly distributed around the segmental equators, numbering 49 at segment VII, 72 at segment XX, and 10 between male pores at segment XVIII. Setal formula is AA:AB:ZZ:ZY = 1:1:2:1 at XIII. Single female pore on the ventral side at segment XIV. Prostomium epilobic. First dorsal pore in 12/13. Clitellum annular in XIV–XVI with no dorsal pores or setae.

Male pores paired on the ventral side at XVIII, in a setal line ~ 5 mm and 0.18× the circumference apart ventrally. Male pores superficial, convex, and surrounded by four genital markings each. Large spermathecal pores three pairs in 6/7–8/9. The distance between each pair ~ 0.27× the circumference ventrally apart, with no genital markings on this area.

The septa at 5/6–7/8 thick, absent in 8/9–9/10, slightly thick in 10/11–11/12, thin in 12/13–14/15, and very thin behind 15/16. Gizzard large in IX–X. Intestine begins in segment XV. Long and simple intestinal caeca in XXVII–XXII. Esophageal hearts four pairs in segment X–XIII. Holandric; testes and funnels in X and XI. Seminal vesicles are paired, one in XI, the other one in XII–XIII. The prostate glands are well developed, located in XV–XXII, and divided into several lobules. The prostate duct is long and slender, surrounded by a large, long sessile glandular mass on the body wall on segments XVII–XIX.

Ovaries located in segment XIII. Three pairs of spermathecae present on VII–IX. Spermathecae large oval sacs of the ampulla, with stout and short duct, it is less than ampullar in length. Diverticulum long and zigzagged at the beginning and dilated towards the distal end, a baton-like chamber.

##### Variation.

Twenty-nine paratypes range in body length from 134–238 mm (197.33 ± 24.52 mm), with 85–162 (142.96 ± 21.25) segments (Table 2).

**Table 2.** Holotype and paratype dimensions and other morphological characteristics of *Amynthassakonnakhonensis* sp. nov.

Characters	Body length (mm)	Number of segments	Location of genital markings	Number of genital markings	First dorsal pore	Prostate glands	Intestinal caeca	Genital marking gland
Holotype CUZM 3823	217	156	XVIII	4	12/13	XV–XXII	XXVII–XXII	present
Paratype CUMZ 3824								
1	190	156	XVIII	4	12/13	XV–XXII	XXVII–XXI	present
2	238	154	XVIII	4	12/13	XV–XXII	XXVII–XXI	present
3	204	150	XVIII	4	12/13	XVI–XXII	XXVII–XXII	present
4	183	156	XVIII	4	12/13	XV–XXI	XXVII–XXII	present
5	172	154	XVIII	4	12/13	XV–XXII	XXVII–XXII	present
6	192	158	XVIII	4	12/13	XV–XXII	XXVII–XXI	present
7	184	153	XVIII	4	12/13	XV–XXIII	XXVII–XXI	present
8	232	153	XVIII	4	12/13	XV–XXII	XXVII–XXI	present
9	201	152	XVIII	4	12/13	XV–XXI	XXVII–XXI	present
10	217	157	XVIII	4	12/13	XV–XXIII	XXVII–XXI	present
11	144	110	XVIII	4	12/13	XV–XXIII	XXVII–XXI	present
12	134	85	XVIII	4	12/13	XV–XXII	XXVII–XXII	present
13	199	159	XVIII	4	12/13	XV–XXII	XXVII–XXI	present
14	217	156	XVIII	4	12/13	XV–XXII	XXVII–XXI	present
15	204	127	XVIII	4	12/13	XV–XXII	XXVII–XXI	present
16	226	159	XVIII	4	12/13	XV–XXII	XXVII–XXI	present
17	208	109	XVIII	4	12/13	XV–XXII	XXVII–XXI	present
18	196	112	XVIII	4	12/13	XV–XXII	XXVII–XXII	present
19	212	156	XVIII	4	12/13	XV–XXII	XXVII–XXI	present
20	191	158	XVIII	4	12/13	XV–XXII	XXVII–XXI	present
21	166	112	XVIII	4	12/13	XV–XXII	XXVII–XXI	present
22	198	127	XVIII	4	12/13	XV–XXI	XXVII–XXI	present
23	176	121	XVIII	4	12/13	XVI–XXIII	XXVII–XXI	present
24	209	162	XVIII	4	12/13	XV–XXII	XXVII–XXII	present
25	193	150	XVIII	4	12/13	XV–XXI	XXVII–XXII	present
26	225	158	XVIII	4	12/13	XV–XXI	XXVII–XXI	present

##### Etymology.

The name *sakonnakhonensis* was derived from the province Sakon Nakhon.

##### Distribution.

This species is known only from the type locality.

##### Remarks.

*Amynthassakonnakhonensis* sp. nov. is sexthecal with spermathecal pores in 6/7–8/9. This species keyed to the *sieboldi* species group (Sims and Easton 1972). After that, Easton (1981) moved *A.sieboldi* (Horst, 1883) to the genus *Metaphire*. Then James et al. (2005) investigated and proposed the *aelianus* species group after *A.aelianus* (Rosa, 1892), to replace the *sieboldi* species group name (James et al. 2005; Bantaowong et al. 2014).

The *aelianus* species group consists of more than 60 species (Sims and Easton 1972; Tsai et al. 1999, 2010; Shen et al. 2003; James et al. 2005; Blakemore 2011; Bantaowong et al. 2014). In the following, we compared the new species with regional species in the *aelianus* species group: *A.osmastoni* (Michaelsen, 1907) from Myanmar, *A.burchardi* Michaelsen, 1899 from Sumatra, *A.monsoonus* James, Shih & Chang, 2005 and *A.huangi* James, Shih & Chang, 2005 from Taiwan (James et al. 2005).

*Amynthassakonnakhonensis* sp. nov. is similar to *A.osmastoni* from Myanmar and *A.burchardi* from Sumatra in the body size, but its easily distinguished by the new species having no median genital markings on segment VIII while *A.osmastoni* has them, the new species having no mid-ventral group of small circular papillae on segment XVIII while *A.burchardi* has them. *Amynthassakonnakhonensis* sp. nov. can be distinguished from *A.monsoonus* and *A.huangi* from Taiwan in that the new species has a larger body size than these two species, *A.monsoonus* has genital markings in segments VII–IX. *Amynthasmonsoonus* and *A.huangi* have no genital markings in the male pores region, whereas the new species contains these characters.

In Thailand, only four species within this species group have been reported from northeastern Thailand. These are *A.fucosus* (Gates, 1933), *A.siam* Blakemore, 2011, *A.arenulus* Bantaowong & Panha, 2014, *A.longicaeca* Bantaowong & Panha, 2014. *Amynthassakonnakhonensis* sp. nov. is easily distinguished from *A.fucosus* from Burma and *A.siam* from Thailand because these species have body diameters of 6 mm and 3 mm, respectively, while the new species are wider at ~ 8–10 mm. *Amynthasfucosus* has two pairs of genital markings at 17/18 and 18/19, and *A.siam* has a single pair between the male pores, while the new species has four genital markings surrounded by male pores. This new species differs from *A.arenulus* in that the latter has a larger size and lacks genital markings in the male pore area. *Amynthasarenulus* has a large transverse elliptical disc surrounded by an elevated rim with an indistinct male aperture located at the outer edge of each poropore, while the new species has four genital markings surrounding the male pore area. The new species has body dimensions similar to *A.longicaeca* but can be distinguished from *A.longicaeca* by the fact that *A.longicaeca* has small crescent-shaped genital markings in the male pore region and spermathecae consist of large oval ampullar with short duct, diverticulum has a small ovate knob and nephridia are present on diverticulum, while the new species has none of these characters.

*Amynthassakonnakhonensis* sp. nov. is similar to *A.bantanensis* sp. nov. in body size but can be distinguished by the *A.sakonnakhonensis* sp. nov. having large oval sacs of the ampulla, with stout and short duct, with long and zigzag diverticulum, and has large, long sessile glandular mass on the body wall region of XVII–XIX, as opposed to *A.bantanensis* sp. nov., which has elongated sacs of the ampulla with slender and long stalks, and has no such glandular mass. *Amynthassakonnakhonensis* sp. nov. differs from *A.auriculus* sp. nov. by the latter has a bit larger size, male pores located in between small three genital markings, each male pore region somewhat ear-shaped after fixation, and consists of a large, elongated ampullar with very short and zigzag diverticulum; however, *A.sakonnakhonensis* sp. nov. has four genital markings surrounding the male pore area, and has a large oval sacs of the ampulla, with long and zigzag diverticulum. *Amynthassakonnakhonensis* sp. nov. different genital marking pattern from the other new *Amynthas* species here, and the other related species is presented in Table 5.

#### ﻿ Amynthas auriculus

Taxon classificationAnimaliaCrassiclitellataMegascolecidae

Chanabun & Panha, sp. nov.

5926B02C-E589-58D1-ABE1-3CEB838E456E

https://zoobank.org/2CA10F55-D79F-4D5C-972F-22FA890B6340

**Figure 7.**
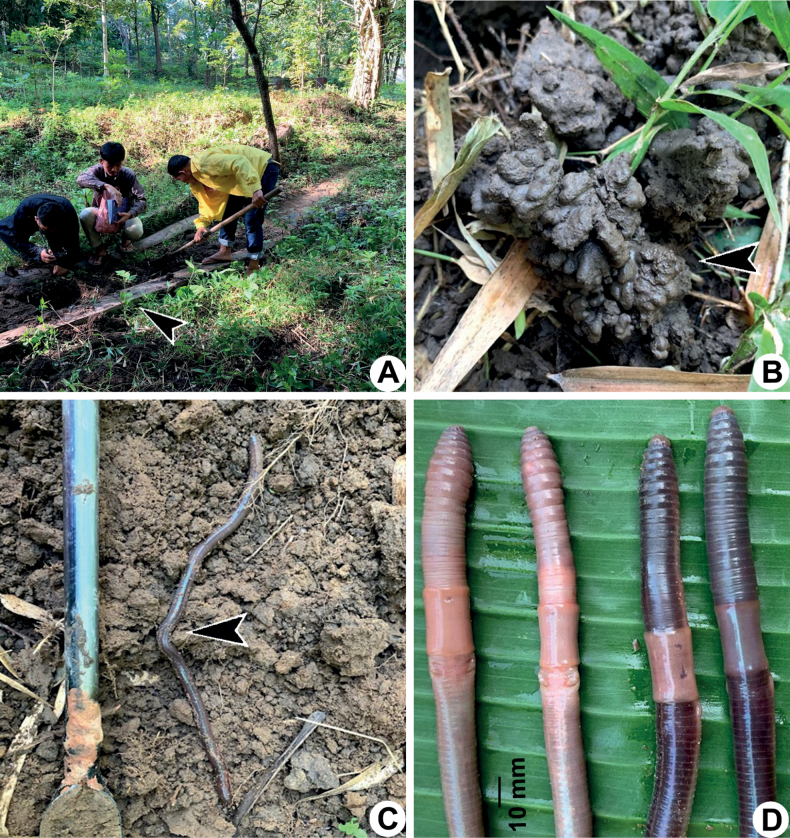
Photographs showing the **A** type locality of *Amynthasauriculus* sp. nov. from Wut Tham Kham, Phannanikom, Sakon Nakhon, Thailand **B** casts of the new species **C** coloration of a living paratype, and **D** coloration of a newly collected paratype (CUMZ 3826) after the first preservation step in 30% (v/v) ethanol.

**Figure 8.**
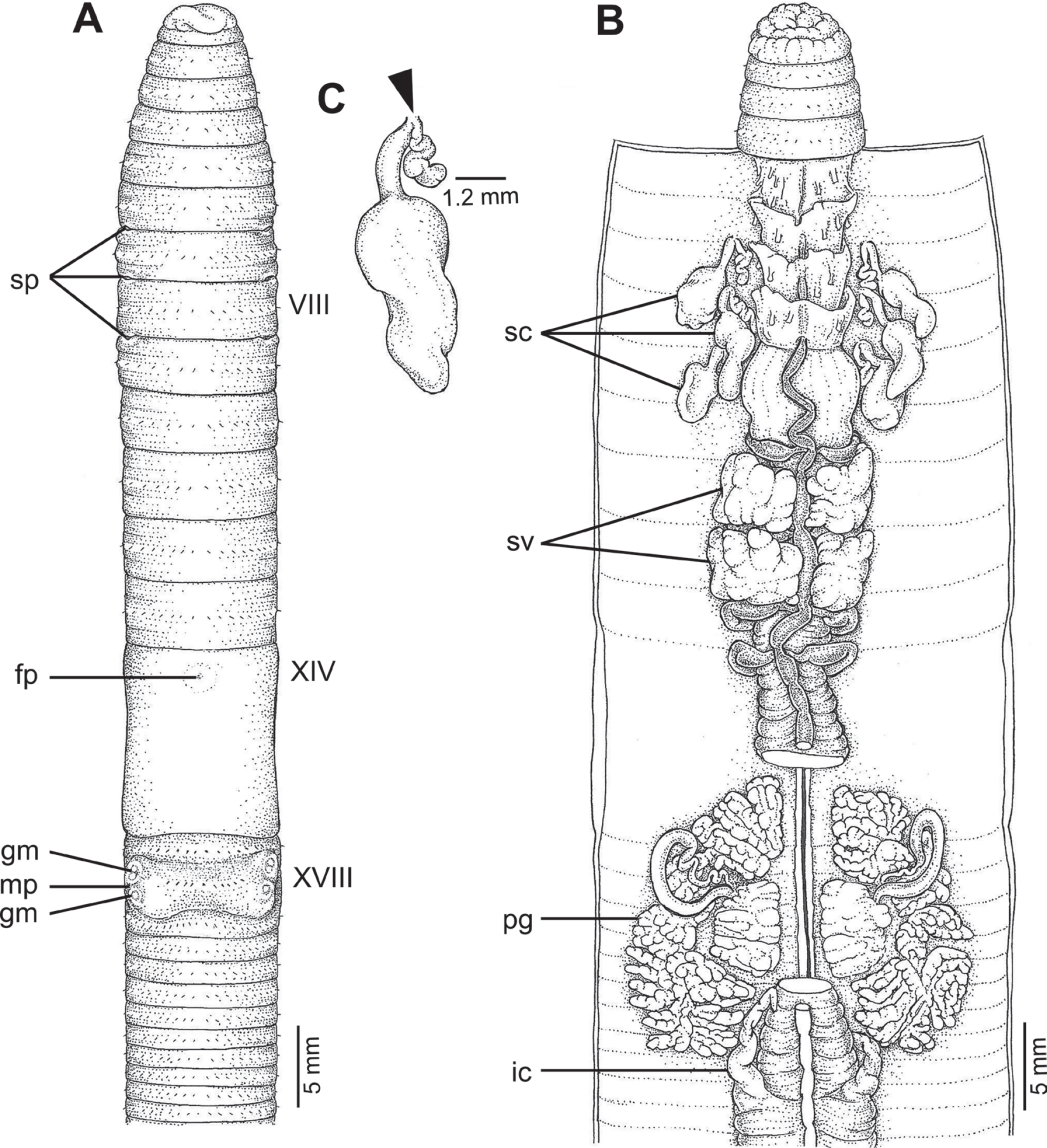
External and internal morphology of *Amynthasauriculus* sp. nov., holotype (CUMZ 3825) **A** external ventral view **B** internal dorsal view, and **C** spermatheca with location of spermathecal pore arrowed.

Figs 7 , 8 , Tables 3 , 5

##### Type material.

***Holotype***: Adult specimen (CUMZ 3825) Wut Tham Kham, Phannanikom, Sakon Nakhon, northeast of Thailand, 17°13'06.2"N, 103°54'00.7"E, 403 m a.m.s.l., 9 October 2019, coll. R. Chanabun, A. Aoonkum. ***Paratypes***: 2 adults (CUMZ 3826), 2 adults (NHMUK), same collection data as for holotype.

##### Diagnosis.

Medium-large size, length 184–267 mm, diameter 9–11 mm, 95–151 segments. Paired male pores at segment XVIII, surrounded by three genital papillae, each male pore region somewhat ear-shaped after fixation. Paired spermathecal pores in intersegments 6/7–8/9. Spermathecae are large, elongated sacs, with slender stalks of the ampulla, and diverticulum very short and zigzagged from its origin until the end. Holandric. Intestinal caeca simple. First dorsal pore in 12/13. Prostate glands large in XVI–XXIV, the prostate duct large, slender, smooth, and a bit zigzagged at the end, surrounded by a large sessile glandular mass on the body wall.

##### Description of the holotype.

Length 267 mm, diameter 10 mm, body cylindrical. 139 segments. Preserved specimens are dark brownish on the dorsal part and pale gray on the ventral part. Setae are regularly distributed around segmental equators, numbering 27 at segment VII, 62 at segment XX, and 20 between male pores at segment XVIII. Setal formula AA:AB:ZZ:ZY = 1:1:2:1 at XIII. At segment XIV, a single female pore mid-ventrally. Prostomium epilobic. First dorsal pore in 12/13. Clitellum annular in segments XIV–XVI with no dorsal pores or setae.

Male pores paired, superficial, convex, located on ventral side of segment XVIII, in between small genital markings; each male pore region somewhat ear-shaped after fixation in ethanol. Male pores ~ 7 mm apart, distance 0.29× the body circumference. Single female pore at XIV. At segment XVIII, each male pore is lined by two anterior genital markings above the setal line, and one posterior genital marking below the setal line. Spermathecal pores three pairs in 6/7–8/9, distance between each pair is ~ 0.37× the circumference ventrally, there are no genital markings in this area.

The septa are thick in 5/6–7/8; absent in 8/9–9/10; slightly thick in 10/11–11/12; thin in 12/13–14/15; and very thin behind 15/16. Gizzard large at IX–X. Intestine begins at segment XV. Long and simple intestinal caeca in XXVII–XXI. Esophageal hearts four pairs in segment X–XIII. Holandric; testes and funnels in X and XI. Seminal vesicles paired in XI–XII. Prostate glands divided into several lobules, in XVI–XXIV, large and well-developed. Prostate duct large, slender, smooth, and a bit zigzagged, surrounded by a large sessile glandular mass on the body wall on segments XVIII–XXI.

Ovaries in segment XIII. Three pairs spermathecae in VII–IX. Spermathecae large, elongated sacs with slender stalks of the ampulla, diverticulum very short and zigzagged from its origin until the end.

##### Variation.

Four paratypes range in size from 184–224 mm (218.20 ± 30.80 mm) body length with 95–151 (128.40 ± 22.65) segments (Table 3).

**Table 3.** Holotype and paratype dimensions and other morphological characteristics of *Amynthasauriculus* sp. nov.

Characters	Body length (mm)	Number of segments	Location of genital markings	Number of genital markings	First dorsal pore	Prostate glands	Intestinal caeca	Genital marking gland
Holotype CUZM 3825	267	139	XVIII	3	12/13	XVI–XXIV	XXVII–XXI	present
Paratype CUMZ 3826								
1	224	151	XVIII	3	12/13	XV–XXV	XXVII–XXI	present
2	207	116	XVIII	3	12/13	XVI–XXIV	XXVII–XXI	present
3	209	141	XVIII	3	12/13	XVI–XXIV	XXVII–XXI	present
4	184	95	XVIII	3	12/13	XVI–XXIII	XXVII–XXI	present

##### Etymology.

The species was named after the region of the male pore that looks like an ear after being preserved.

##### Distribution.

This species is known only from the type locality.

##### Remarks.

*Amynthasauriculus* sp. nov. belongs to the *aelianus* species group. This new species is quite similar to *A.osmastoni* from the South Andaman Islands and *A.burchardi* from Sumatra in the body dimensions but differs in having genital markings in the male pore area and having large sessile glandular masses on the body wall in XVIII–XXI, while *A.osmastoni* has genital markings in the spermathecal pores region without glandular masses on the body wall, and *A.burchardi* has a group of circular papillae in mid-ventral XVIII with no glandular masses on the body wall. *Amynthasauriculus* sp. nov. can be distinguished from *A.monsoonus* and *A.huangi* from Taiwan in which the new species has a larger body size, *A.monsoonus* has a body size of 102 mm, a diameter of 3.6 mm, with 83 segments and genital markings in segments VII–IX, while the new species is absent. *Amynthashuangi* has a body size of 70 mm, a diameter of 3.5 mm, with 101 segments, and genital marking glands in the body wall in VI–IX, while the new species has no such character. *Amynthasmonsoonus* and *A.huangi* have no genital markings in the male pores region, whereas the new species is present.

*Amynthasauriculus* sp. nov. is easily distinguished from the other four earthworms of the *aelianus* species group reported from Thailand, namely *A.fucosus* from Myanmar, *A.siam* from Thailand, by its size and other physical traits. *Amynthasfucosus* has a body size range of 120 mm with a diameter of 6 mm and 114 segments, *A.siam* has a body size range of 73 mm, diameter of 3 mm, while the new species has a body size that ranges from 184–267 mm with a diameter of 9–11 mm, and 95–151 segments. *Amynthasfucosus* has two pairs of genital markings at 17/18, 18/19, and *A.siam* has single pair between the male pores, while the new species has three genital markings in the male pores. This new species differs from *A.arenulus* in that *A.arenulus* has no genital markings. In contrast, the new species has them at segment XVIII, near the male pores area. The new species is relatively similar to *A.longicaeca* in body size; however, *A.longicaeca* differs in that it has an oval ampulla, a small ovate knob with nephridia on the diverticulum, and also has a crescent-shaped male pore region (Table 5).

#### ﻿ Amynthas bantanensis

Taxon classificationAnimaliaCrassiclitellataMegascolecidae

Chanabun & Panha, sp. nov.

A3313BAC-959B-5628-8AB3-A21F97D37878

https://zoobank.org/10F71AA3-5917-4F80-846F-A0683B6063AC

**Figure 9.**
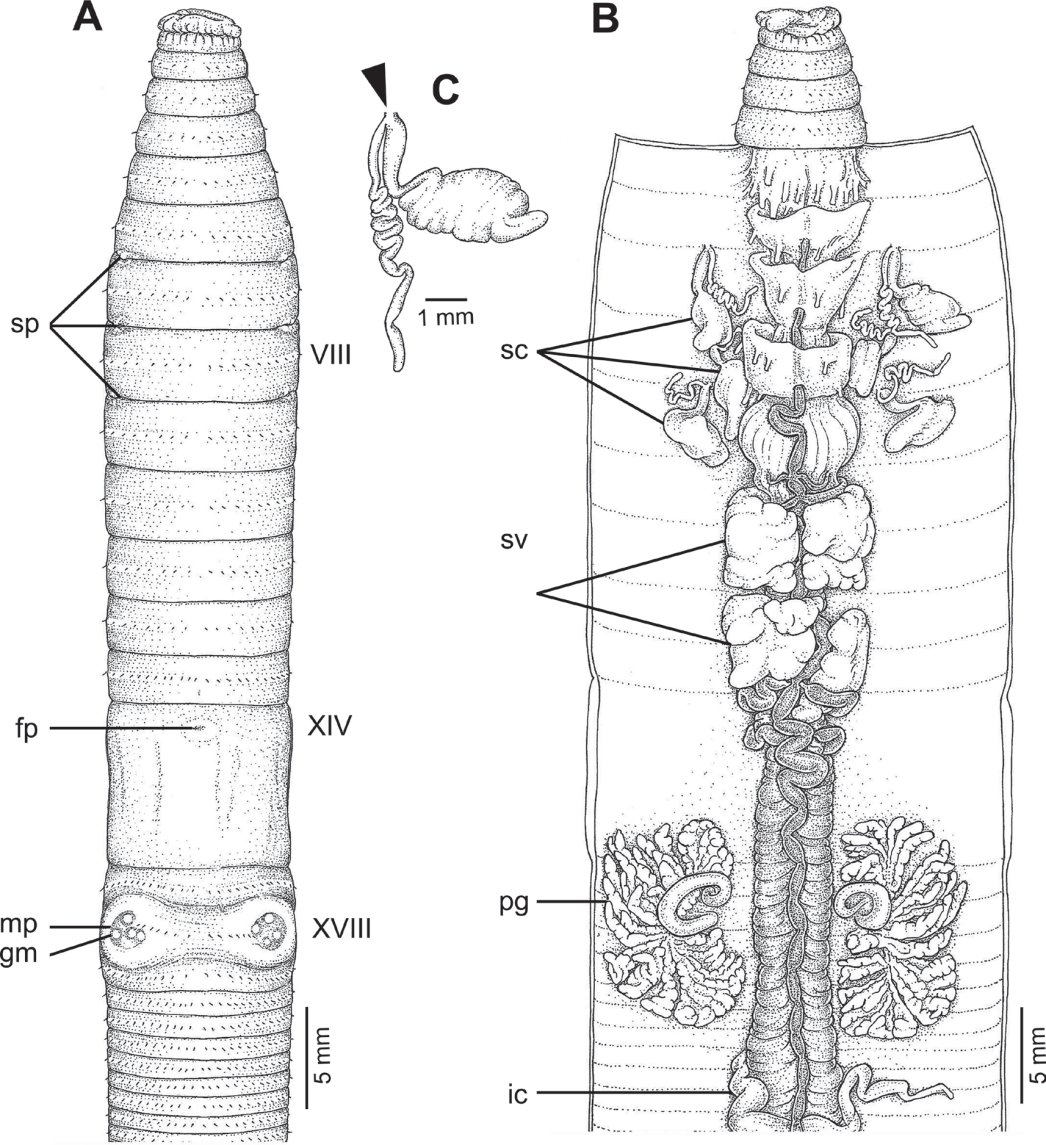
External and internal morphology of *Amynthasbantanensis* sp. nov., holotype (CUMZ 3827) **A** external ventral view **B** internal dorsal view, and **C** spermatheca with location of spermathecal pore arrowed.

Fig. 9 , Tables 4 , 5

##### Type material.

***Holotype***: Adult specimen (CUMZ 3827) Ban Tan, Nawah, Nakhon Phanom, northeast of Thailand, 17°30'28.0"N, 104°05'48.0"E, 149 m a.m.s.l., 28 September 2018, coll. R. Chanabun, A. Aoonkum. ***Paratypes***: 3 adults (CUMZ 3828), 2 adults (NHMUK); same collection data as for holotype.

##### Diagnosis.

Medium-large size, length 159–213 mm, diameter 8–10 mm, 105–149 segments. Paired male pores in segment XVIII, surrounded by four genital papillae each. Paired spermathecal pores in intersegments 6/7–8/9. Spermathecae elongated sacs, with slender and long stalks of the ampulla, diverticulum slender, long at the beginning, zigzagged at the center, and slender towards the end. Holandric. Intestinal caeca are simple. First dorsal pore in 12/13. Prostate gland large and well developed in XVI–XXII; its duct is short, smooth and curled like a spiral.

##### Description of holotype.

Length 191 mm, diameter 8 mm, body cylindrical, 127 segments. Preserved specimens are dark brownish on the dorsal part and pale gray on the ventral part. Setae are regularly distributed around the segmental equators, numbering 37 at segment VII, 57 at segment XX, and 12 between male pores at segment XVIII. Setal formula AA:AB:ZZ:ZY=1:1:2:1 at XIII. Single female pore mid-ventrally at segment XIV. Prostomium epilobic. First dorsal pore in 12/13. Clitellum annular in XIV–XVI with no dorsal pores or setae.

Male pores paired on the ventral side of XVIII, ~ 9 mm apart, distance 0.32× body circumference. Male pores superficial, convex, each surrounded by four genital markings. Spermathecal pores three pairs in 6/7–8/9. The distance between each pair is ~ 0.41× the body circumference ventrally apart. There are no genital markings in this area.

The septa in 5/6–7/8 thick, absent at 8/9–9/10, slightly thick in 10/11–11/12, thin in 12/13–14/15, and very thin behind 15/16. Gizzard large in IX. Intestine begins at segment XV. Vary long and simple intestinal caeca in XXVII–XIX. Esophageal hearts four pairs in segment X–XIII. Holandric; testes and funnels in X and XI. Seminal vesicles are paired, one in X–XI, the other one in XII–XIII. The prostate glands are large and well-developed, located in segments XVI–XXII and divided into several lobules. Prostate duct is relatively short, smooth, and spiraled.

Ovaries in segment XIII. Three pairs spermathecae in VII–IX. Spermathecae elongated sacs, with slender and long stalks of the ampulla, diverticulum slender, long at the beginning, zigzagged at the center, and slender towards the end.

##### Variation.

Five paratypes range in size from 159–213 mm (183.83 ± 20.54 mm) body length with 105–149 segments (124 ± 14.39 mm) (Table 4).

**Table 4.** Holotype and paratype dimensions and other morphological characteristics of *Amynthasbantanensis* sp. nov.

Characters	Body length (mm)	Number of segments	Location of genital markings	Number of genital markings	First dorsal pore	Prostate glands	Intestinal caeca	Genital marking gland
Holotype CUZM 3827	191	127	XVIII	4	12/13	XVI–XXII	XXVII–XIX	absent
Paratype CUMZ 3828								
1	179	105	XVIII	4	12/13	XV–XXII	XXVII–XIX	absent
2	213	149	XVIII	4	12/13	XV–XXIII	XXVII–XIX	absent
3	159	124	XVIII	4	12/13	XV–XXII	XXVII–XIX	absent
4	164	119	XVIII	4	12/13	XV–XXII	XXVII–XX	absent
5	197	120	XVIII	4	12/13	XV–XXIII	XXVII–XIX	absent

##### Etymology.

The name *bantanensis* is given to this species for its type locality at Ban Tan, Nawah, Nakhon Phanom.

##### Distribution.

This species is known only from the type locality.

##### Remarks.

*Amynthasbantanensis* sp. nov. is sexthecal with spermathecal pores in 6/7–8/9, belonging to the *aelianus* species group. The new species differs from other *aelianus* species group reported by body size, location of spermathecae and spermathecae, *A.bantanensis* sp. nov. is similar to *A.burchardi* from Sumatra, and *A.osmastoni* from the south Andaman Islands in body dimensions, but easily distinguished from *A.burchardi* which has a mid-ventral group of small circular papillae on segment XVIII, while they are absent in the new species. *Amynthasosmastoni* has genital markings at the pre-clitellum, which are absent in the new species. *Amynthasbantanensis* sp. nov. differs from *A.monsoonus* and *A.huangi* from Taiwan in that the new species has genital markings in the male pores region, whereas they are absent in *A.monsoonus* and *A.huangi*. *Amynthasmonsoonus* has a smaller body size of 102 mm, a diameter of 3.6 mm, with 83 segments, and genital markings in segments VII–IX. *Amynthashuangi* has a smaller body size, a body range of 70 mm, a diameter of 3.5 mm, and 101 segments, and *A.huangi* has genital marking glands in the body wall in VI–IX, while the new species lacks them. The new species has genital markings in the male pores region, whereas they are absent in *A.monsoonus* and *A.huangi*.

*Amynthasbantanensis* sp. nov. differs from *A.arenulus* from Surin, Ubon Ratchathani, and Srisaket in that *A.arenulus* has a large transverse elliptical disc surrounded by an elevated rim with an indistinct male aperture located at the outer edge of each poropore and consists of a large sessile glandular mass on the body wall in this region, while the new species has genital markings at segment XVIII, near the male pore, but has no glandular mass on the body wall. *Amynthasbantanensis* sp. nov. is easily distinguished from *A.fucosus* from Burma and *A.siam* from Thailand because both previously reported species have smaller body sizes (*A.fucosus* = 6 mm, *A.siam* = 3 mm). *Amynthasfucosus* has two pairs of genital markings at 17/18, 18/19, and *A.siam* has a single pair between the male pores, while the new species has four genital markings surrounded by male pores at segment XVIII. The new species, *Amynthasbantanensis*, is similar body dimensions to *A.longicaeca* from Chaiyaphum, but is easily distinguished by *A.longicaeca* having crescent-shaped genital markings and a large sessile glandular mass on the body wall, while the new species lacks it. Considering the differences between *A.bantanensis* sp. nov., *A.auriculus* sp. nov., and *A.sakonnakhonensis* sp. nov. see the respective remarks sections above and Table 5.

**Table 5.** Morphological characteristics of *A.sakonnakhonensis* sp. nov., *A.auriculus* sp. nov., *A.bantanensis* sp. nov., and other *aelianus* species in Thailand, Burma, Sumatra. The comma is used to separate body length and width. Missing data are shown with a question mark (?). Data for *A.arenulus* Bantaowong & Panha, 2014, *A.longicaeca* Bantaowong & Panha, 2014, *A.burchardi* Michaelsen, 1899, *A.osmastoni* (Michaelsen, 1907), and *A.fucosus* (Gates, 1933) are from Bantaowong et al. (2014), and *A.siam* Blakemore, 2011 is from Blakemore (2011).

Characters	*A.sakonnakhonensis* sp. nov.	*A.auriculus* sp. nov.	*A.bantanensis* sp. nov.	*A.arenulus*	*A.longicaeca*	*A.burchardi*	*A.osmastoni*	*A.fucosus*	*A.siam*
Body length (mm), width (mm)	134–238, 6–9	184–267, 9–11	159–213, 8–10	465, 13.3	278, 10.1	270, 9	250–320, 10–11	120, 6	73, 3
Segment number	85–162	95–151	105–149	133–176	115–160	126	126–148	114	?
First dorsal pore	12/13	12/13	12/13	12/13	12/13	13/14	12/13	12/13	12/13
Genital marking									
Pre-clitellum	absent	absent	absent	absent	absent	absent	median in VIII	absent	absent
Post-clitellum	four genital markings at XVIII	three genital markings at XVIII	four genital markings at XVIII	absent	crescent shape	mid-ventral group of small circular papillae on XVIII	absent	paired at 17/18, 18/19	pair at XVIII
Spermathecae	large oval sac	large elongated sacs	elongate sac	large sac	oval	oval	spherical	flatten	spherical
Diverticulum	long and zigzag, distal end a baton-like chamber	short and zigzag	slender, long, at the beginning, zigzag at the center and slender toward the end	slender	small ovate knob	long slender	tubular	tubular and coiled	convoluted
Prostate gland	XV–XXII	XVI–XXIV	XVI–XXII	XVII–XXII	XVI–XXI	XV–XX	XV–XXIV	XVII–XX	?
Intestinal caeca	XXVII–XXII	XXVII–XXI	XXVII–XIX	XXVII–XXIII	XXVII–XXIV	?	?	?	XXVII–?
Genital marking gland	large, long sessile glandular mass on segment XVII–XIX	large sessile glandular masses on segment XVIII–XXI	absent	large sessile glandular masses	large sessile glandular masses	absent	absent	absent	slight sessile genital glands
Type locality	Thailand	Thailand	Thailand	Thailand	Thailand	Sumatra, Indonesia	South Andaman Islands	Myanmar	Thailand

## ﻿Discussion

The benefits of earthworms have led to a variety of uses, such for decomposing organic waste from agriculture and households, as well as to enhance soil qualities and structure (Alegre et al. 1996; Sharma et al. 2017) and produce the best compost for plant growth and organic farming systems (Rajiv et al. 2009). Moreover, earthworms are used as food for animals. In some local areas, they serve as human food and a component of herbal medicine since the earthworm’s tissue is rich in nutrients and enzymes that are very good for health (Grdiša et al. 2013; Sun and Jiang 2017), as mentioned in the introduction section.

In this paper, four new earthworm species are reported: one species of genus *Metaphire* and three species of genus *Amynthas*, of which three species were collected by locals and dried for sale (*Metaphiresongkhramensis* sp. nov. from Sakon Nakhon, *Amynthassakonnakhonensis* sp. nov. from Sakon Nakhon, and *Amynthasbantanensis* sp. nov. from Nakhon Phanom). *Metaphiresongkhramensis* sp. nov. belongs to the *houlleti* species group. This new species has the longest and widest body size among the *houlleti* groups reported in Thailand. *Amynthassakonnakhonensis* sp. nov., *A.auriculus* sp. nov., and *A.bantanensis* sp. nov. are classified in the *aelianus* species group. All of these new species were reported from northeast Thailand and collected from different habitat types.

*Metaphiresongkhramensis* sp. nov. lives in the dark clay soil of the oxbow lake of the Songkhram River, which locals call Kud Klong Ngong. This habitat is covered with plenty of earthworm casts and with mainly small bamboo (*Bambusa*sp., *Bambusaarundinacae* Wild.), small shrubs (*Hymenocardiawallichii* Tul. and *Euryeomalongifolia* Jack.), and trees (*Syzygiumcumini* L. and *Lagerstroemiacalyculata* Kurz.). The earthworms move around on the soil, but, interestingly, all of them are the same species.

On the other hand, Nong Tuet, Sakon Nakon, from where *Amynthassakonnakhonensis* sp. nov. was found, is a wetland. Water will flood in the rainy season and be covered by grass in the dry season. Villagers in the area use it as a pasture for livestock, such as cows and buffaloes. This habitat’s soil is a mixture of clay and tiny pebbles, and the land could not be utilized for cultivation because shrubs predominate the area. The authors found that the new species of earthworms inhibit this area together with *M.peguana* (Rasa, 1890) and *Drawida*sp.

*Amynthasauriculus* sp. nov. lives in dark sandy loam soil covered with a mixed deciduous forest at Wut Tham-kham, Sakon Nakon. This area is a fertile forest of the Phu Phan Mountain range. Contrastingly, *Amynthasbantanensis* sp. nov. lives in the sandy loam of the soil covered with casts. This species was collected early in the morning from the field where rice and other economic plants are cultivated.

Furthermore, after interviewing the locals, the authors learned that every August to December of the year, from the end of the rainy and cold seasons (temperature ranges from 16–22.9 °C), earthworms come out from the soil at approximately 2:00–5:00 a.m. At this time the locals collect them for sale. If the weather is excellent, 30–80 kg/person of earthworms can be collected, but if it is otherwise rainy, very cold, or windy, the earthworms do not come out. Moreover, these earthworms also choose to stay underground during the full moon.

## Supplementary Material

XML Treatment for Metaphire songkhramensis

XML Treatment for Amynthas sakonnakhonensis

XML Treatment for Amynthas auriculus

XML Treatment for Amynthas bantanensis

## ﻿Additional information

### Author ORCIDs

Ratmanee Chanabun https://orcid.org/0000-0003-4584-2773

Anuwat Aoonkum https://orcid.org/0000-0002-3895-9570

Teerapong Seesamut https://orcid.org/0009-0003-9589-8641

Ueangfa Bantaowong https://orcid.org/0009-0000-6068-7930

Somsak Panha https://orcid.org/0000-0002-4431-2458

### Data availability

All of the data that support the findings of this study are available in the main text.
